# Epulis: A Narrative Review of Epidemiology, Clinical Features, Pathogenesis, and Management

**DOI:** 10.3290/j.ohpd.c_2258

**Published:** 2025-09-03

**Authors:** Wenli Gu, Simin Li, Shaonan Hu, Yanmei Yao, Xiao Jiang, Deborah Kreher, Gerhard Schmalz, Liyi Jiang, Wenxia Meng

**Affiliations:** a Wenli Gu Attending Physician, Stomatological Hospital, School of Stomatology, Southern Medical University, S366 Jiangnan Boulevard, Haizhu District, Guangzhou City, Guangdong Province, China. Conducted the initial literature search and wrote the manuscript.; b Simin Li Attending Physician, Stomatological Hospital, School of Stomatology, Southern Medical University, S366 Jiangnan Boulevard, Haizhu District, Guangzhou City, Guangdong Province, China. Performed additional literature searches, participated in drafting discussions, and manuscript revision.; c Shaonan Hu Attending Physician, Stomatological Hospital, School of Stomatology, Southern Medical University, S366 Jiangnan Boulevard, Haizhu District, Guangzhou City, Guangdong Province, China. Performed additional literature searches, participated in drafting discussions, and manuscript revision.; d Yanmei Yao Head Nurse, Stomatological Hospital, School of Stomatology, Southern Medical University, S366 Jiangnan Boulevard, Haizhu District, Guangzhou City, Guangdong Province, China. Performed additional literature searches, participated in drafting discussions, and manuscript revision.; e Xiao Jiang Associate Chief Physician, Stomatological Hospital, School of Stomatology, Southern Medical University, S366 Jiangnan Boulevard, Haizhu District, Guangzhou City, Guangdong Province, China. Performed additional literature searches, participated in drafting discussions, and manuscript revision.; f Deborah Kreher Attending Physician, Department of General Medicine, University of Leipzig, Germany. Performed additional literature searches, participated in drafting discussions, and manuscript revision.; g Gerhard Schmalz Department Director, Department of Conservative Dentistry and Periodontology, Brandenburg Medical School (MHB) Theodor Fontane, Brandenburg an der Havel, Germany. Served as the senior author, critically reviewed the manuscript for scientific accuracy and clinical relevance, and guided the conceptual framework.; h Liyi Jiang Chief Physician, Stomatological Hospital, School of Stomatology, Southern Medical University, S366 Jiangnan Boulevard, Haizhu District, Guangzhou City, Guangdong Province, China. Administered and supervised the whole research project.; i Wenxia Meng Department Director, Stomatological Hospital, School of Stomatology, Southern Medical University, S366 Jiangnan Boulevard, Haizhu District, Guangzhou City, Guangdong Province, China. Administered and supervised the whole research project.

**Keywords:** epulis, gingival reactive hyperplastic lesions, fibrous epulis, vascular epulis, pregnancy epulis, giant cell epulis, peripheral giant cell granuloma

## Abstract

**Purpose:**

Epulis represents a group of reactive hyperplastic lesions occurring in the gingival area, distinct from true hypertrophy, as these lesions involve tissue proliferation rather than enlargement of existing cells. These lesions present significant clinical challenges in diagnosis and management.

**Methods:**

This narrative review synthesises current literature on epulis, examining epidemiological patterns, clinical characteristics, pathogenic mechanisms, and treatment approaches across different subtypes.

**Results:**

Epulis primarily affects young and middle-aged adults with female predilection. The molecular pathogenesis involves complex interactions between local irritants and signalling pathways, particularly aryl hydrocarbon receptor (AhR) and RAS-PI3K-AKT-NF-κB activation. Definitive diagnosis requires histopathological examination, with fibrous, vascular, and giant cell variants exhibiting distinctive features. Surgical excision remains the primary treatment, though emerging evidence supports laser therapy, sclerotherapy, and combination approaches. Preventing recurrence necessitates elimination of local irritants, regular periodontal maintenance, and awareness of patient-specific risk factors.

**Conclusions:**

Clinicians should perform thorough clinical and radiographic examinations to differentiate epulis from malignancies, and consider subtype-specific management strategies, implement comprehensive prevention protocols, and conduct long-term follow-up, especially for high-risk cases. Future research should focus on developing targeted molecular therapies, standardised treatment guidelines, and personalised recurrence prevention strategies.

Epulis refers to reactive hyperplastic lesions occurring in the gingiva (gingival reactive hyperplastic lesions, GRHL), classified as oral/gingival reactive lesions. Clinically, it presents as usually painless pedunculated or sessile masses, varying in colour from light red to red, and in appearance from non-ulcerated flat lesions to ulcerated masses. The size ranges from a few millimetres to several centimetres.^29^ These localised overgrowths of reactive gingival hyperplastic lesions are not neoplasms.^29,35^ Epulis affects a wide age range, with patients aged 1–98 years, peaking between 30–60 years. The prevalence varies from 5.6% to 20.6%,^61,62,128,141,183^ with a higher incidence in females.^29,183^ Epulis originates from periodontal tissues, with fibrous and vascular types primarily caused by chronic local irritants such as plaque, calculus, defective restorations, and ill-fitting prosthetics. Giant cell epulis follows a distinct aetiology involving complex pathological processes, including inflammatory responses and multinucleated giant cell formation. Hormonal factors, particularly elevated oestrogen and progesterone, contribute significantly to pregnancy-associated epulis, which must be differentiated from medication-induced gingival enlargements caused by anticonvulsants (phenytoin), immunosuppressants (cyclosporine), and calcium channel blockers (nifedipine).

Epulis is a common oral lesion that can cause various effects, including bleeding, chewing and speech dysfunction. When occurring in the anterior dental region, it may affect aesthetics and even cause severe psychological issues.^59^ The diagnosis and treatment of epulis present clinical challenges. Although epulis is benign, it often recurs. Different studies report varying recurrence rates, with overall rates ranging from 2.9% to 15.2%,^21,62,171,183^ and multiple recurrences (≥2 times) accounting for 11.11–16% of recurrent cases.^21,183^ Treatment of epulis includes surgical excision with scalpel, laser, and electrosurgery, along with the removal of potential causes through plaque and calculus removal, replacement of defective restorations, and elimination of traumatic habits. For most types of epulis, regular oral hygiene maintenance and professional follow-up can reduce the recurrence rate.^144^

According to the 2018 new classification of periodontal and peri-implant diseases,41 epulis is categorised as a reactive lesion of non-plaque-induced gingival diseases, including fibrous epulis, calcifying fibroblastic granuloma, vascular epulis (pyogenic granuloma (PG)), and peripheral giant cell granuloma (PGCG). Currently, the most widely accepted and common histological classification of epulis includes these four subtypes.^22,183^ The incidence of different types of epulis varies among regions and populations. Although the aetiology seems similar, each subtype produces different clinical and histological changes,^22^ with variations in treatment methods and prognosis, recurrence rates. Therefore, classification is crucial for developing treatment plans.

Several critical knowledge gaps persist in epulis research and management: (1) incomplete understanding of molecular pathogenesis mechanisms limiting targeted therapy development; (2) absence of large-scale multicentre epidemiological studies across diverse populations; (3) insufficient comparative data on long-term efficacy of various treatment modalities; (4) limited evidence supporting preventive strategies; (5) unexplored relationships between systemic conditions and epulis development; and (6) lack of standardised management guidelines, particularly for special populations. This narrative review aims to synthesise current knowledge on epulis epidemiology, clinical features, pathogenesis, and management approaches, while identifying areas of consensus and controversy to guide future research and improve patient outcomes.

## METHODS

### Fibrous Epulis

#### Epidemiology

Fibrous epulis is an inflammatory fibrous hyperplastic nodule of the gingiva. As shown in Table 1, it has various synonyms, including fibroepithelial polyp (FEP), fibroma, focal fibrous hyperplasia (FFH), inflammatory fibrous hyperplasia (IFH), irritation fibroma (IF), or traumatic fibroma (TF).^24,33,49,56,82^ When caused by biting, it may be termed a biting fibroma (BF). Fibroma is most common on the buccal mucosa, followed by the tongue, lips, hard palate, and gingiva in decreasing frequency.^49^ As illustrated in Table 1, fibrous epulis occurs across a wide age range but is more common in females,^22,33,183^ possibly due to the role of female hormones in promoting fibroblast proliferation and collagen accumulation.^56^ Recent studies by Zhao et al^183^ reviewing 2,971 epulis cases in China over 12 years found fibrous epulis to be the most common histological subtype at 60.92%. This aligns with Baesso et al^22^’s report of 47% fibrous epulis among 996 cases in Brazil over 8 years. However, other studies have reported frequencies as low as 13%,^127^ with literature reporting a range of 13–68%,^22, 61,106,127,183^ as summarised in Table 1. These variations may be due to differences in case numbers, terminology, diagnostic criteria, geographic regions, and genetic factors.

**Table 1:** Clinical and demographic features of various types of epulis, including fibrous epulis, vascular epulis, and giant cell epulis. The table presents a comprehensive comparison of these epulis subtypes, detailing their synonyms, frequencies, gender predilection, age distribution, common sites of occurrence, characteristic colours, typical size ranges, and recurrence rates.

Epulis	Synonyms	Frequencies	Gender	Age	Site	Colour	Size	Recurrence rate
Fibrous epulis	fibroepithelial polyps (FEP), fibroma, focal fibrous hyperplasia (FFH), inflammatory fibrous hyperplasia (IFH), irritation fibroma (IF), traumatic fibroma(TF),biting fibroma (BF)^24,49,56,82,127^	13–68%^22,61, 106,127,183^	More common in females^22,33,183^	Across a wide age range	The maxilla is more common than the mandible^22,183^ and the anterior region is more common than the posterior region^22,183^	Usually matches the colour of the surrounding gingiva but may appear as a localised ulcer^56,107,129^	A few millimetres to several centimetres^25,82,129^ typically less than 1.5 cm, rarely exceeding 3 cm^.82,107^	9.55%^183^
Vascular epulis	PG of the gingiva, lobular capillary haemangioma, or telangiectatic granuloma^33,158,112^	8.08–57%^22, 62,183^	More common in females^112,153,154^	Across a wide age range, most prevalent in the 20–30 years^119,158^	The maxilla is more common than the mandible,^22,146^ and the anterior region is more common than the posterior region.^146^ The mandible is more common than the maxilla, and the posterior region is more common than the anterior region^183^	From pink to deep red or reddish-purple^15,126^	A few millimetres to several centimetres, rarely exceeding 2.5 cm^95^	17.18%^183^
Giant cell epulis	peripheral giant cell granuloma (PGCG), peripheral giant cell reparative granuloma^33,47^	1–47%^22,83,127^	Most studies indicate a higher prevalence in females^47^ although some reports suggest a higher incidence in males^12,61^	Across a wide age range, most prevalent in the 40–60 years^4^	The mandible is more common than the maxilla,^4,22,176,183^ and the posterior region is more common than the anterior region^183^	Usually red-purple, sometimes appearing blue to brown^56^	A few millimetres to several centimetres, typically less than 2 cm, although occasionally may exceed 4 cm^53^	8.82%^183^


#### Clinical presentation and differential diagnosis

**Fig 1a to d:**
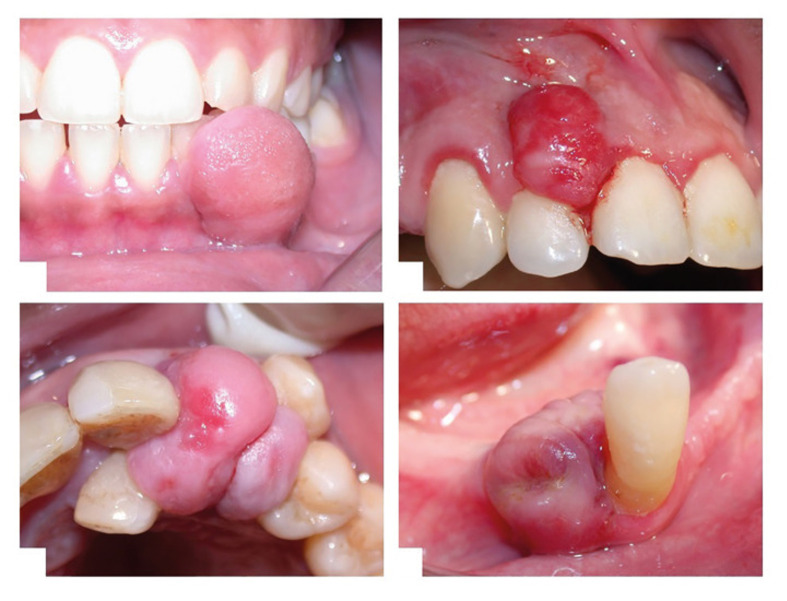
Clinical presentation of fibrous hyperplasia (a), pyogenic granuloma (b), peripheral ossifying fibroma (c), and peripheral giant cell lesion (d).

As summarised in Table 1 and shown in Figure 1,^22^ fibrous epulis presents as an exophytic, smooth, pedunculated or sessile fibrous mass attached to the gingiva. It usually matches the colour of the surrounding gingiva, but may appear as a localised ulcer. Table 1 indicates that the diameter can reach several centimetres,^25,82,129^ typically less than 1.5 cm, rarely exceeding 3 cm.^82,107^ It is usually asymptomatic, with only 7.8% (15 out of 193 cases) reported as painful.^56^ As shown in Table 1 and Figure 1,^22^ fibrous epulis most commonly occurs interdentally, and it may cover the tooth surface. When large, it can extend across the contact point to both sides of the dental arch, appearing dumbbell-shaped – although this appearance is more typical of giant cell epulis.^33^ In denture wearers, similar lesions may occur due to ill-fitting dentures, termed denture-induced fibrous hyperplasia or denture epulis.^90^ Extensive gingival overgrowth may also suggest hereditary gingival fibromatosis, which can occur as an isolated lesion or as part of a syndrome.

#### Histology and histological diagnosis

**Fig 2a to l:**
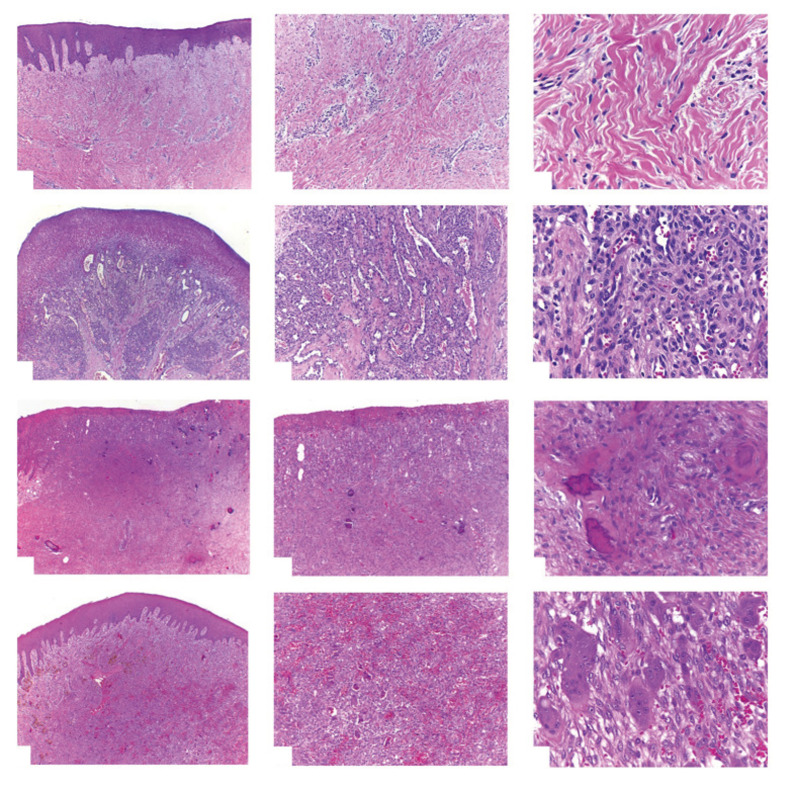
Haematoxylin and eosin (HE)-stained sections from the studied lesions. Inflammatory fibrous hyperplasia showing a fibrous proliferation covered by a stratified squamous epithelium (a, HE 40×) and details of the fibrous component permeated by a mild chronic inflammatory infiltrate (b, HE 100× and c, HE 400×). PG showing an ulcerated surface covered by a fibrin membrane (d, HE 40×), and granulation tissue composed by inflammatory cells, small blood vessels, and fibroblasts (e, HE 100× and f, HE 400×). Peripheral ossifying fibroma showing an ulcerated surface (g, HE 40×), and a proliferation of mesenchymal spindle cells associated with areas of calcified tissue (h, HE 100× and i, HE 400×). Peripheral giant cell lesion characterised by the presence of a vascularised tissue with deposition of haemosiderin (j, HE 40×), and details of the haemorrhagic areas associated with the presence of multinucleated giant cells and mononuclear cells (k, HE 100× and l, HE 400×).

As presented in Figure 2,^22^ histologically, fibrous epulis consists of dense collagen fibre bundles with proliferating and usually keratinised squamous epithelium or ulceration on nodular fibrous connective tissue. Chronic inflammatory cells (such as plasma cells and lymphocytes) may infiltrate the lesion.^183^ The degree of collagenisation and vascularity depends on the maturity and presence of inflammation. Fibroblasts are typically small and spindle-shaped in most lesions. However, in some cases, fibroblasts may be large, stellate-shaped, occasionally multinucleated, with thin, elongated epithelial projections. These lesions are termed ‘giant cell fibroma’ (GCF),^61,106,114^ most commonly found on the tongue and gingiva of young individuals.

According to pioneers in the study of oral mucosal fibrous overgrowth, Barker and Lucas,^25,49,107^ irritation fibromas exhibit two patterns of collagen arrangement: radiating and circular, depending on the site of the lesion and the amount of irritation. The former occurs in sites where the mucosa is immobile over bone (such as the palate) when the trauma is greater, while the latter is induced by lesser trauma and occurs in movable sites not fixed to bone (such as the buccal mucosa).

Up to one-third of gingival lesions contain metaplastic bone trabeculae, especially in the labial gingiva of the maxilla. This lesion is termed peripheral ossifying fibroma (POF) (Figure 1^22^) (synonyms: calcifying fibroblastic granuloma, peripheral ossifying fibroma, ossifying fibroid epulis). POF is identified by fibrous tissue proliferation and varying degrees of mineralisation. As seen in Figure 2,^22^ histologically, the mineralised component consists of trabeculae or droplet-like calcifications resembling woven and lamellar bone, osteoid-like material, or dystrophic calcification in an active cellular matrix background.^33,183^ This lesion has a higher recurrence rate than other forms of fibrous epulis.^34,134^ POF occurs exclusively on the gingiva, most commonly in the anterior maxillary region, and is more frequent in young females.^19^ The lesion diameter is typically less than 1.5 cm but can be larger, rarely causing separation of adjacent teeth and alveolar ridge resorption.^34,82,134^ However, as the lesion size increases over time, it may occasionally present with bone surface erosion or even destruction.^166^

### Treatment Modalities

#### Scalpel surgical excision and removal of stimulating factors

Fibrous epulis primarily originates from the gingival connective tissue or periodontal ligament. As shown in Table 2, surgical excision is the preferred treatment method for FFH,^56,58,131^ with narrow margin excision being the choice.^24^ Additionally, eliminating the cause, scaling adjacent teeth to remove irritants, and minimising recurrence are crucial.^107^ Zhao et al^183^ reported a recurrence rate of 9.55% for FFH, lower than PG (17.18%) and POF (12.98%), but higher than PGCG (8.82%). Multiple recurrences of epulis can be attributed to failure to eliminate causal factors (such as persistent irritation and trauma, and incomplete surgical excision) and genetic regulation.^103^ Santos et al^56^ and Sambhashivaiah^149^ suggest that FFH is unlikely to recur unless the inciting trauma persists or repeats. Lalchandani et al^107^ reported a case of recurrent irritation fibroma on the palatal side of a 13-year-old boy’s maxillary incisor, successfully managed by re-excision and wearing a removable anterior bite plane to initiate orthodontic management of deep bite. Follow-up at 9 months showed no recurrence of the BF, indicating the importance of eliminating irritants or causes in preventing fibroma recurrence.

**Table 2:** Summary of current treatment modalities for Focal Fibrous Hyperplasia (FFH). This table provides a comprehensive overview of various therapeutic approaches used in the management of FFH, including surgical excision, laser therapies, and combination treatments. Each treatment modality is presented alongside the corresponding authors and publication years, offering a chronological perspective on treatment evolution

	Treatment modalities	Authors, year
1	Surgical excision with a scalpel	Santana et al (2014),^56^ Lalchandani et al (2020)^107^
2	Complete excision and soft tissue augmentation	Salaria et al (2021)^147^
3	Diode laser surgery	Sangle et al (2024),^150^ Do Amaral et al (2023),^58^ Oliveira et al (2017)^55^ Kohli et al (2016)^102^ Gupta et al (2015),^78^ Pai et al (2014),^131^ Eliades et al (2010)^64^
4	Diode laser surgery and the scalpel surgery	Çayan et al (2019),^39^ Bakhtiari et al (2015),^24^ Amaral et al (2015),^11^ Ayoub et al (2014)^20^
5	Diode laser surgery versus electrocautery	Jesus et al (2020)^54^
6	CO_2_ laser excision	Suter et al (2014),^165^ Suter et al (2017)^164^
7	Er:YAG excision	Suter et al (2017)^164^
8	Nd:YAG excision	Ibrahim et al (2023),^85^ Vescovi et al (2010)^172^


#### Laser therapy

Besides scalpel excision, Table 2 illustrates other methods, including electrosurgery, cold scalpel, and various types of laser surgery.^122^ Lasers used for oral soft tissue lesion excision include CO_2_ lasers, erbium lasers (Er:YAG and Er,Cr:YSGG), Nd:YAG, and diode lasers.^39,113,130^ Conventional surgery has complications such as intra- and postoperative bleeding, difficult wound healing, deep anaesthesia, swelling, scarring, and postoperative pain.16,20 Diode lasers have become popular due to their small size and ease of use for minor oral soft tissue surgeries.^130,163^ They offer advantages such as reduced bleeding, scarring, pain, infection, swelling, shortened operation time, and good hemostasis.^131,132,163,124^ Therefore, some literature reports that lasers are more effective in reducing bleeding and pain compared to traditional surgery, electrosurgery, and cryosurgery in excising irritated fibromas.^16,20, 24,39,64^ Regarding the impact on pathological diagnosis of fibroepithelial hyperplasia, Monteiro et al^122^ and Tenore et al^169^ reported that when used correctly, electrosurgical scalpels and lasers do not limit or hinder histopathological diagnosis. Furthermore, Monteiro et al^122^ suggest adding 1–2 mm to healthy tissue (extending margins depending on laser wavelength) when using laser excision to reduce the thermal effect on lesion margins. Research on laser treatment of fibrous epulis is mostly case reports or small case series. Large-cohort, multicentre, standardised, and long-term follow-up studies are still needed to further clarify the therapeutic effects of laser excision of IFH, such as the impact of different parameter settings and lesion sizes on postoperative healing, and the time and rate of recurrence after laser excision.^39^

#### Removal of stimulating factors

Additionally, some scholars^49^ suggest that for patients with younger lesions, if the source of irritation is removed, irritation fibromas may spontaneously regress. This is supported by evidence based on fluorescence and polarised light microscopic evaluation of collagen arrangement in irritation fibromas. Compared to older lesions, collagen in younger irritation fibromas is less organised and may regress without additional treatment if the source of irritation is eliminated. Therefore, if the lesion is newly acquired, conservative treatment (including complete removal of the irritation-related fibroma) can be considered as an initial intervention.

#### Complete excision and soft tissue augmentation for FFH

For large or recurrent epulis cases, extensive surgical excision may lead to large residual soft tissue defects, causing postoperative discomfort, root exposure sensitivity, aesthetic issues, and difficulty in maintaining oral hygiene.^42,80,148^ Salaria et al^147^ reported a case of simultaneous repair of soft tissue defects after excision of recurrent gingival irritation fibroma using a coronally repositioned flap (CRF), with good soft tissue defect coverage at 9 months postoperatively. The authors consider CRF to be planned according to the basic requirements of the attached gingival strip, soft tissue defect, and deep vestibule. Moreover, CRF is time-efficient, easy to learn and perform, and the coronally advanced flap (CAF) can be used alone,^170^ or in combination with platelet-rich fibrin (PRF)148 or other materials with good results.^45^ Currently, case reports on simultaneous repair of soft tissue defects after excision of irritation fibromas are still limited,^147^ but there are increasingly more similar research reports.

#### Complete excision and soft tissue augmentation for POF

Regarding the excision of POF, Salaria et al^146^ employed a modified laterally positioned flap (LPF) to repair a gingival defect following POF surgical removal. This approach aligns with the report by El Ayachi et al,^19^ who similarly used a laterally displaced flap (LDF) to treat gingival defects after POF excision, achieving stable postoperative results without recurrence. Previous literature reported various surgical techniques for treating mucosal gingival defects, including subepithelial connective tissue graft (SCTG), CAF, LDF, free gingival graft (FGG), and PRF. These techniques can be used individually or in combination.^19,8^ For instance, Walters et al^174^ utilised three reconstructive surgical procedures – a LPF, a SCTG, and a coronally positioned flap – to repair gingival defects following POF excision, achieving satisfactory restorative outcomes. Chitsazha et al,^44^ Chaudhari et al,^42^ and Hutton et al^84^ used free gingival FGG to manage post-POF excision gingival defects, obtaining good functional and aesthetic results. Addressing gingival defects through periodontal plastic surgery concurrently with the excision of gingival epulis can prevent and reduce postoperative complications such as functional impairments.

### Vascular Epulis

#### Epidemiology

As summarised in Table 1, vascular epulis, also known as PG of the gingiva, lobular capillary haemangioma, or telangiectatic granuloma,^33,158,112^ is a reactive vascular lesion rather than a true granuloma or infection-related condition. It is primarily caused by trauma or repeated irritation, and is also associated with hormonal changes during puberty, pregnancy, or oral contraceptive use.^33^

Table 1 indicates that oral PG can occur not only on the gingiva but also on other oral mucosal surfaces including the cheek, lip, tongue, and palate (except for the floor of the mouth).^5,112^ However, it primarily manifests on the gingiva, accounting for 75% of all cases.^72,112,129^ As shown in Table 1, the lesions are slightly more prevalent on the maxillary gingiva compared to the mandibular gingiva, with anterior teeth being more susceptible than posterior teeth. Buccal lesions are more common than lingual lesions.^112^ While PG can affect individuals of all age groups, with reported cases ranging from 4.5 to 93 years old, it is most prevalent in the 20–30 age bracket.^119,158^ Furthermore, a higher incidence is observed in females.^112,153,154^ In a study by Zhao et al^183^ involving 2,971 cases of epulis in China, PG accounted for 8.08% of cases, ranking third after FFH (60.92%) and POF (29.32%), but higher than PGCG (1.68%). Similarly, Baesso et al^22^ reported that among 996 cases of epulis in Brazil, PG represented 28% of cases, second only to FFH (47%), and higher than POF (18%) and peripheral giant cell lesion (PGCL) (7%). However, some studies^6,95, 121,160^ have reported PG as the most common subtype. The literature indicates that the frequency of PG ranges from 8.08% to 57%.^22,62,183^ This wide variation may be attributed to differences in sample size, terminology, diagnostic criteria, geographical location, and genetic factors among different populations.

#### Clinical presentation and differential diagnosis

As described in Table 1 and Figure 1,^22^ PG presents as a soft, smooth or lobulated exophytic mass, which may be pedunculated or sessile. Epivatanos et al^65^ reported that lobular capillary haemangioma (LCH) type PG more frequently occurs as sessile lesions (66%), while non-LCH PG tends to be pedunculated (77%). These lesions are prone to spontaneous bleeding or bleeding upon minor trauma and may be ulcerated. Table 1 and Figure 1^22^ illustrate that the colour of PG ranges from pink to deep red or reddish-purple, depending on the duration of the lesion and the degree of vascularisation, which also indicates the extent of vascularisation and fibrosis.^15,126^ According to Table 1, the size of granulomatous epulis varies from a few millimetres to several centimetres, rarely exceeding 2.5 cm. These lesions typically reach their maximum size within weeks or months, after which they remain stable indefinitely.^32^ While they generally grow slowly, asymptomatically, and painlessly,^28^ rapid growth can occur in some cases.^133^ Table 1 also summarises the common sites of PG occurrence.PG is more common on the labial and buccal aspects of the gingiva compared to the lingual or palatal sides and can affect both sides of the gingiva, including interdental areas.^154^ Granulomatous epulis rarely causes significant bone loss and typically does not present radiographic features.^96,119^ However, most case reports in the literature lack radiographic analysis. Some authors have reported bone loss or alveolar ridge erosion in areas associated with PG.^112,115^ Angelopoulos^14^ also noted that in certain cases, long-standing gingival PG can lead to localised alveolar bone resorption. In cases of extensive gingival vascular lesions, it is less likely to be a vascular epulis or PG. Instead, systemic causes of gingival vascular dilation should be considered, such as leukaemia or granulomatosis with polyangiitis (GPA). The latter typically presents orally as ‘strawberry gingivitis’.^33^

#### Histology and histological diagnosis

As illustrated in Figure 2,^22^ histopathologically, granulomatous epulis is primarily composed of granulation tissue with inflammatory cells (such as neutrophils, lymphocytes, and plasma cells), accompanied by endothelial cell proliferation, capillary hyperplasia, and minimal fibrous tissue. The lesion is covered by a thin layer of ulcerated squamous epithelium.^183^ PG is histologically classified into two types: LCH type and non-LCH type^.89,116^ The LCH type is characterised by lobular aggregation of blood vessels, without specific changes such as oedema, capillary dilation, or inflammatory granulation tissue reaction. The non-LCH type consists of a vascular core similar to granulation tissue with focal fibrous tissue. Compared to the non-LCH type, the LCH type exhibits a greater number of small-lumen diameter vessels in the lobular areas.^73^ According to the classification by the International Society for the Study of Vascular Anomalies (ISSVA, 2022), some PG (also referred to as lobular capillary haemangiomas) are categorised as vascular tumours. Sato et al^152^ histologically described the majority of oral PG as the LCH type.

### Pregnancy Epulis

Pregnancy-associated epulis, also known as pregnancy epulis or pregnancy tumours, is predominantly vascular in nature. These lesions, occurring during pregnancy, are referred to as pregnancy granuloma, PG of pregnancy, or granuloma gravidarum (GG).^43^ The incidence of GG ranges from 1.7% to 8.5%.^1,2,43,52,60,135^ The development of GG is associated with chronic low-grade irritation (such as plaque, calculus, or trauma) and elevated hormone levels. These lesions can occur at any stage of pregnancy. Oestrogen and progesterone levels peak in late pregnancy, affecting local tissues and their microvascular systems. Oestrogen increases vascular endothelial growth factor in macrophages, leading to enhanced gingival vasodilation and lesion growth.^74,135^ Progesterone’s effects are more pronounced than those of oestrogen.^99^ Consequently, pregnancy tumours reach their peak in the middle to late stages of pregnancy, with the lowest proportion occurring in the first trimester.^38,52,175^ Furthermore, female sex hormone levels influence the microecology of plaque biofilms,^69^ potentially increasing the risk of gingival tumour formation. GG typically present as red, soft masses that may ulcerate due to secondary trauma and are prone to bleeding. They grow rapidly and vary in size, usually not exceeding 2 cm in diameter.^43^ These lesions primarily occur on the gingiva, most commonly on the labial and buccal aspects of the maxilla.^151,156^ Patients with GG usually complain of bleeding, painless swelling, impaired masticatory function, and aesthetic concerns. From a histopathological perspective, there is no distinction between pregnancy granuloma and PG.^38^ The diagnosis of GG or pregnancy tumour is based on aetiology and the apparent influence of female sex hormones.^161^ In cases of rapid growth with irregular alveolar bone resorption, pregnancy tumours should be differentiated from oral squamous cell carcinoma (OSCC).^43^ While this differential diagnosis is important, it’s worth noting that OSCC is rare in young pregnant women. Nevertheless, clinicians should remain vigilant as there are similarities in clinical and radiographic presentations between the two conditions, although they differ significantly in histopathology. OSCC often presents intraorally as a lobulated granulomatous lesion, potentially with an ulcerated surface, which can lead to misdiagnosis of some OSCC cases as epulis.^30^

#### Treatment of GG

Treatment of pregnancy tumours depends on specific circumstances, with treatment plans formulated based on the severity of the epulis, the pregnant woman’s overall condition, and foetal development. Treatment of pregnancy tumours emphasises early diagnosis and intervention, including non-surgical periodontal treatment and surgical treatment, with non-surgical treatment (ie, basic periodontal treatment) being the first choice.^43^ First, pregnant women should receive oral hygiene education and guidance on oral hygiene techniques and correct brushing methods. In many cases, pregnancy epulis often only requires management during pregnancy, as these lesions tend to regress spontaneously after delivery due to hormonal stabilisation. This natural regression may eliminate the need for surgical intervention, particularly if local irritants are removed.^135^ The focus of treatment during pregnancy should be on maintaining good oral hygiene and managing any symptoms.

Postpartum, if the epulis persists, a re-evaluation should be conducted to determine if further treatment is necessary. This approach acknowledges the often self-limiting nature of pregnancy epulis while ensuring appropriate follow-up care. Therefore, under permissible systemic conditions, Cheng et al^43^ and Zhang et al^182^ believe that regardless of the stage of pregnancy, not limited to the second trimester (4–6 months), basic periodontal treatment should be received as early as possible to eliminate local stimulating factors. However, this approach must be considered critically, as the potential risks to the unborn child should always be taken into account, especially during the first trimester. While basic periodontal treatment is generally considered safe, the timing and extent of any dental intervention during pregnancy should be carefully evaluated on a case-by-case basis. The first trimester, in particular, is a critical period for foetal development, and elective procedures are often postponed until later in pregnancy. Any treatment plan should be made in consultation with the patient’s obstetrician, balancing the need for oral health management with the paramount concern for foetal safety. However, care should be taken to ensure gentle operations, shorten treatment time, and treat in stages or sections as appropriate.

Considering that oral PG is very common in pregnant women (in about half of the cases), excision of pregnancy tumours during pregnancy may lead to recurrence of the lesion at the same site on the gingiva in subsequent pregnancies.^105,173^ Therefore, if the lesion is small, painless, non-bleeding, and does not affect occlusal function, clinical observation and follow-up can be considered;^2,27,173^ furthermore, observation may be extended until postpartum, with surgical treatment deferred, provided that the tumour does not continue to enlarge or impact occlusal function after basic periodontal treatment has been administered.^43^ Iorio et al^87^ concluded after retrospective analysis that the clinical management of pregnancy tumours depends on the type and severity of symptoms, determined by bleeding, pain, and bone loss shown on radiographs. For epulis with persistent pain or infection that interferes with oral function or continues to enlarge, timely surgical excision is necessary,^76,173,185^ and differential diagnosis should be noted. The ideal time for dental treatment is between the 17th and 28th week of pregnancy.^67^ If necessary, surgical treatment can be completed in the second trimester (4–6 months) with postpartum follow-up,^8,135^ but intervention should be timely after risk assessment if the tumour severely affects the pregnant woman’s life. If the lesion does not resolve after delivery, surgical treatment is still needed. Additionally, plaque control is very important to prevent posttreatment recurrence.^38^ Traditional epulis surgical excision is more traumatic and causes more bleeding. Therefore, Cheng et al^43^ reported using the Nd:YAG laser to excise pregnancy tumours, which has the advantages of being minimally invasive, comfortable, haemostatic, and bactericidal. However, care should be taken to follow standard procedures to prevent excessive irradiation leading to necrosis and damage of the alveolar bone and other tissues.^48^ For larger pedunculated tumours, ligation can be used before excision to block the blood supply and reduce tumour size.^43^

### Treatment Modalities

As summarised in Table 3, the current treatment options for PG are diverse and include various approaches. These treatment modalities, as detailed in Table 3, encompass removal of causative stimulating factors, surgical excision, electrocautery, laser therapy, cryosurgery, corticosteroid injection, and sclerotherapy.^158^ While Table 3 presents a comprehensive overview of these treatments, traditional surgical treatment remains the primary approach. The following sections will discuss each of these treatment modalities in detail, expanding on the information provided in Table 3 and highlighting their respective advantages, limitations, and clinical applications.

**Table 3:** Comprehensive overview of current treatment modalities for pyogenic granuloma (PG). This table presents a wide range of therapeutic approaches used in the management of PG, including minimally invasive techniques, surgical excision, various laser therapies, sclerotherapy, and combination treatments. Each treatment method is listed alongside the corresponding authors and publication years, providing a chronological perspective on the evolution of PG management strategies

	Treatment modalities	Authors, year
1	Minimally invasive	Chandrashekar et al (2012),^40^ Frumkin et al (2015)^71^
2	Surgical excision with a scalpel	Noaman et al (2020)^10^
3	Complete excision and soft tissue augmentation	Bosco et al (2006),^31^ Joda (2012),^93^ Salaria et al (2018),^148^ Güler et al (2024)^77^
4	Electrocautery	Shirbhate et al (2024)^154^
5	Diode laser surgery	Asnaashari et al (2015),^17^ Al-Mohaya et al (2016),^9^ Pisano et al (2021),^136^ Andreadis et al (2019)^13^
6	Diode laser surgery and the scalpel surgery	Isola et al (2018),^88^ Karci et al (2017)^98^
7	CO_2_ laser excision	Lindenmüller et al (2010)^110^
8	Er:YAG excision	Fekrazad et al (2014),^68^ Reza et al (2014)^142^
9	Nd:YAG excision	Yadav et al (2018),^178^ Zeng et al (2020)^180^
10	Flashlamp-pumped pulsed dye laser treatment	Wu et al (2022)^176^
11	Pingyangmycin sclerotherapy	Cai et al (2017)^37^
12	Injection of absolute ethanol	Ichimiya et al (2004)^86^
13	Injection of the ethanolamine oleate solution	Matsumoto et al (2001),^118^ Sayed et al (2022),^63^ Khaitan et al (2018)^100^
14	Sodium tetradecyl sulphate	Soni (2021),^159^ Khaitan et al (2018),^100^ Shivhare et al (2019),^155^ Deore et al (2014)^57^


#### Removal of stimulating factors

For small, painless, non-bleeding PG, some scholars have proposed minimally invasive treatment. Chandrashekar^40^ reported a case of gingival PG treated with minimally invasive therapy, which involved scaling and root planning of the affected tooth for four consecutive weeks, monitoring the lesion’s progress weekly. If the lesion persisted, scaling and root planning were performed weekly. After four weeks, the lesion had largely regressed with no recurrence at 6-month follow-up. Frumkin et al^71^ also successfully treated two cases of recurrent gingival PG through non-surgical treatment, including strict oral hygiene instruction, scaling, root planning, and maintenance therapy, with no recurrence at 1–2-year follow-up.

#### Scalpel surgical excision

Surgical excision remains the preferred treatment for PG.^112,154^ The surgical procedure involves excision with a 2 mm margin beyond the lesion edge, including the periosteum, curettage of the periodontal membrane in the corresponding area, removal of supra- and subgingival plaque and calculus, and elimination of stimuli (foreign bodies, sources of trauma, defective restorations, etc.) to prevent recurrence.^10,72,73,117^ Bhaskar et al^157^ reported a recurrence rate of 15.8% after conservative excision. Zhao et al^183^ reported a recurrence rate of 17.18% for PG, higher than POF (12.98%), FFH (9.55%), and PGCG (8.82%). Multiple recurrences of epulis can be attributed to failure to eliminate causative factors (eg, persistent stimulation and trauma, and incomplete surgical excision) and genetic regulation.^103^

Literature on the immediate management of residual gingival defects after excision of reactive gingival lesions is limited.^148^ Salaria et al^148^ reported a case using PRF combined with a CAF to repair residual gingival defects after excision of recurrent PG in the maxillary aesthetic segment, achieving good gingival defect coverage. Joda^93^ and Güler et al^77^ used SCTG to repair defects after gingival PG excision, all achieving good root coverage. Bosco et al^31^ used a LPF to repair a gingival defect in one case of recurrent PG removal in the upper anterior teeth. After 5 years of follow-up, the root coverage was good with no recurrence.

#### Laser therapy

Similar to the excision of other oral soft tissue lesions like fibrous epulis, laser treatment of granulomatous epulis has several advantages, including reduced bleeding, lower postoperative infection rates, faster recovery, less postoperative pain, minimally invasive treatment, no need for sutures, and reduced intra- and postoperative complications. Laser treatments for granulomatous epulis include Er:YAG laser^68,142^ Nd:YAG laser,^178,180^ diode laser,^9,13,17,136^ CO_2_ laser,^110^ and flashlamp-pumped pulsed dye laser.^158,176^ These methods provide more assurance in recurrent/multiple lesions, but further clinical trials are needed.

#### Sclerotherapy

Sclerotherapy involves injecting sclerosing agents into the vascular lumen to selectively eliminate small blood vessels, varicose veins, and vascular malformations. The goal of sclerotherapy is to destroy the vessel wall, turning it into a non-patent fibrous cord.^57^ Sclerosing agents used for granulomatous epulis or oral granulomas include pingyangmycin (bleomycin [BLM] A5 hydrochloride, PYM), anhydrous ethanol, sodium tetradecyl sulphate (STS), and corticosteroids.^37^ Although these sclerosing agents have proven effective, their limitations should not be overlooked.^37^ For instance, the use of ethanol is restricted because some patients are allergic to alcohol, and its injection may cause severe soft tissue oedema.^184^ STS infiltration into the matrix tissue can cause non-specific necrotic changes.^89^ Moreover, local nerve damage has been reported after STS sclerotherapy for venous malformations.^162^

##### PYM injection

The cytotoxicity of PYM is mainly DNA damage. The mechanism of PYM on granulomatous epulis may be promoting endothelial cell apoptosis and inducing endothelial-mesenchymal transition (EndoMT), thereby reducing the number of vascular lumens, thickening the lumen wall, leading to lumen narrowing or occlusion.^37^ Cai et al^37^ reported treating 16 cases of recurrent granulomatous epulis with pingyangmycin injection, with all cases fully recovering without recurrence and no systemic complications. Postoperative complications were local swelling and pain, which resolved without intervention within six days of injection.

##### Ethanol injection

Injection of ethanol causes cell dehydration and necrosis. Anhydrous ethanol is cheaper than other sclerosing agents such as ethanolamine oleate and polyols. Ichimiya et al^86^ reported five cases of PG that recurred after inadequate cryosurgery treated with anhydrous ethanol injection. The lesions were completely removed after 3 weeks, with postoperative complications of pain and swelling, but no other side effects were observed.

##### Ethanolamine oleate injection

Matsumoto et al^118^ reported treating nine cases of PG by local injection of monoethanolamine oleate solution. All lesions were completely removed without recurrence, and scarring was inconspicuous. Only one patient complained of pain due to an avoidable excessive injection of the solution.

##### STS injection

STS sclerotherapy has been widely used for direct sclerosis of varicose veins and endoscopic sclerosis of gastroesophageal varices. Deore et al^57^ reported a case of successful treatment of recurrent oral PG associated with port-wine stain (PWS) by STS injection. Khaitan et al^100^ successfully treated 40 cases of oral PG through consecutive injections of sclerosing agent 1–4 times per week.

Besides causing endothelial cell damage to eliminate vascular lumens, STS may lead to non-specific necrotic changes in the matrix tissue. Adverse reactions to STS treatment include allergic reactions, skin necrosis, and hyperpigmentation. STS injection is usually painless; therefore, extravasation may develop asymptomatically.^112^ To avoid skin necrosis, slow and careful injection with some pressure is required.^123^ Sclerotherapy for PG is a new treatment method, and more case studies are still needed to test its efficacy and safety.

##### Corticosteroid injection

Parisi et al^133^ first reported successful treatment of a case of oral PG that had recurred multiple times after surgical excision through intralesional injection of corticosteroids. Bugshan et al^36^ also treated a case of granulomatous epulis that recurred after multiple surgical excisions, successfully treated by injection of triamcinolone suspension and local application of clobetasol propionate ointment for 2 weeks, with the lesion disappearing after 3 weeks. The exact mechanism of corticosteroid treatment is unclear. Corticosteroids can enhance the response of vascular bed lesions to vasoconstrictors. For example, dexamethasone can inhibit the angiogenic potential of haemangioma-derived stem cells and downregulate pro-angiogenic factors to block vascular proliferation.^75^

### Giant Cell Epulis

#### Epidemiology

As shown in Table 1, giant cell epulis, also termed PGCG or peripheral giant cell reparative granuloma,^33,47^ originates from the periosteum or periodontal membrane following local irritation or chronic trauma. Cases of PGCG developing after traumatic tooth extraction have been reported (4). Table 1 illustrates that while PGCG can occur at any age, it is most common between 40–60 years. Most studies indicate a higher prevalence in females, with a ratio of 1.2:1,47 although some reports suggest a higher incidence in males.^12,61^ The prevalence of PGCG among different types of epulides varies across studies, as reflected in Table 1. Zhao et al^183^ reported that PGCG accounted for only 1.68% of epulides in a Chinese population, while Baesso et al^22^ found PGCG to comprise 7% of epulides in a Brazilian cohort. Conversely, Naderi et al^127^ reported PGCG as the most prevalent lesion (47%). Table 1 summarises these findings, showing that the reported frequency of PGCG ranges from 1% to 47% in the literature.^22,83,127^ This wide variation in prevalence, as illustrated in Table 1, underscores the importance of considering geographical and population differences when interpreting epidemiological data on PGCG.

#### Clinical presentation and differential diagnosis

As summarised in Table 1 and Figure 1,^22^ PGCG typically presents as a well-defined, sessile or pedunculated soft tumour-like protuberance on the gingiva or alveolar mucosa.80 Table 1 and Figure 1^22^ describe the lesion’s characteristic appearance: usually red-purple in colour, sometimes appearing blue to brown. This characteristic blue-purple pigmentation is due to the presence of hemosiderin. As noted in Table 1, PGCG can extend through the contact point between teeth in a dumbbell shape and is more commonly found in the mandible.^4,47^ Generally asymptomatic, approximately one-third of cases exhibit erosion of adjacent bone.^47^ Table 1 indicates that the diameter of PGCG is typically less than 2 cm, although occasionally it may exceed 4 cm.^53^ Clinically, as shown in Table 1, PGCC resembles PG, but compared to typical PG, the former often appears blue-purple in colour. PGCG is more likely to cause bone resorption than PG.^89^ PGCG is a soft tissue lesion that rarely affects the underlying bone, but sometimes the alveolar bone may undergo surface erosion, resulting in a ‘cup-shaped’ superficial resorption.^29^

Differential diagnosis of giant cell lesions includes giant cell tumour (GCT), central giant cell granuloma (CGCG), PGCG, cherubism, aneurysmal bone cyst (ABC), and brown tumour of hyperparathyroidism (BHT).^129,177^ Special examinations such as radiography, calcium, phosphate, alkaline phosphatase, and parathyroid hormone levels can aid in differentiating these lesions.^70^ Among these, the first three are giant cell lesions in oral tumours that require more attention in differential diagnosis. GCT is more prone to recurrence and more invasive than giant cell granulomas (GCG), with a higher rate of malignant transformation compared to CGCG and PGCG.^129^ Malignant transformation and lung metastasis of GCT have been reported.^81^ The recurrence rates presented in Table 1 show that GCG is approximately 10–15% with no associated metastasis, while GCT has a higher local recurrence rate (about 25%) with risks of malignant transformation and metastatic progression.^108^

GCT occurring in the craniofacial region is rare (accounting for 2–7%).^23^ In comparison, GCG is more common in the oral cavity. GCG is divided into CGCG and PGCG, differing from GCT in their unique occurrence in the maxillofacial bones and non-neoplastic nature, often indicating post-traumatic or infectious repair processes.^81^ However, location alone cannot be used to diagnose GCT versus GCG. Although both have similar clinical and radiological presentations, their prognoses and treatment management differ, as shown in Table 1. Central giant cell granuloma, located within the jawbones, exhibits more invasive and aggressive behaviour.^29^ CGCG is of bone origin, accounting for 1–7% of benign maxillofacial bone lesions. PGCG is a variant originating from the gingiva, with an incidence rate 3–5 times that of intraosseous GCG.^26^ Histologically, GCT and GCG have overlapping pathological features and are often difficult to diagnose definitively. Hoarau et al^81^ suggest that the most distinguishing factors are symptoms (particularly pain, reported in 72% of GCTs but only 15% of GCGs) and the distribution pattern of giant cells in the stroma (80% of GCTs show uniform distribution, while 86.7% of GCGs show clustered distribution). In cases where certain factors may point towards GCT, immunohistochemistry and molecular genetics can be used for further diagnostic assistance.^81^

### Histopathology and Diagnosis

As shown in Figure 2,^22^ the histopathological features of PGCG include unencapsulated proliferation of mononuclear spindle and polygonal cells with osteoclast-type multinucleated giant cells in a vascular background.^47^ The presence of multinucleated giant cells distinguishes PGCG from other reactive hyperplasia.^140^ However, PGCG can be histologically difficult to differentiate from other giant cell lesions. In many cases, initial excision may be incomplete, necessitating additional examinations such as radiography and serum calcium level measurements to exclude other giant cell lesions.^33^

### Treatment Modalities

#### Scalpel surgical excision

As shown in Table 4, surgical excision with a scalpel remains the gold standard for treating PGCG.79 Current treatment approaches involve surgical excision combined with curettage or peripheral ostectomy to reduce the risk of recurrence.^47,81,104^ Chrcanovic et al^47^ reported a recurrence rate of 16% for simple excision of PGCG, which decreased to 2.8% with additional curettage and 0% with peripheral ostectomy. Compared to simple excision, excision followed by curettage reduced the recurrence rate by 85%.^47^ In a Chinese study, Zhao et al^183^ reported a recurrence rate of 8.82% for PGCG, lower than that of PG (17.18%), POF (12.98%), and FFH (9.55%). Recurrence of PGCG after traditional scalpel excision can be attributed to the lack of deep excision, including the periodontal ligament.^138^ This may be related to surgical technique, necessitating re-excision.^168^ Notably, studies have reported a high recurrence rate (1/3) for implant-associated PGCG, with recurrence rates unaffected by post-excision curettage. In some cases, removal of the associated implant may be necessary to prevent recurrence.^46,143^

**Table 4:** Comprehensive overview of current treatment modalities for peripheral giant cell granuloma (PGCG). This table presents a range of therapeutic approaches used in the management of PGCG, including surgical excision, laser therapies, and alternative treatments such as sclerotherapy. Each treatment method is listed alongside the corresponding authors and publication years, providing a chronological perspective on the evolution of PGCG management strategies

	Treatment modalities	Authors, year
1	Surgical excision with a scalpel	Chrcanovic et al (2018)^47^
2	Complete excision and soft tissue augmentation	Alaa’Z et al (2014)^6^ Sahingur et al (2004)^145^
3	Diode laser surgery	Dalipi et al (2023),^53^ Pulicari et al (2022)^139^
4	High-level laser therapy	Hanna et al (2023)^79^
5	Ethanolamine oleate sclerotherapy	Ahmed et al (2016)^3^


#### Laser therapy and other treatments

Table 4 also presents alternative treatment modalities for PGCG. Current literature has rarely reported any invasive tendencies or malignant transformation of PGCG.^7,18^ However, Hanna et al^79^ reported successful treatment of an aggressive, rapidly recurring PGCG using high-level laser therapy (HLLT) after three failed surgical excisions, suggesting HLLT as a potential alternative to standard surgical treatment. Dalipi et al^53^ described the excision of a 2 cm PGCG in the anterior mandible using a 975 nm infrared diode laser, highlighting advantages such as reduced bleeding, no need for sutures, and rapid wound healing. However, evidence for laser therapy in PGCG management remains limited.^66,79,143^ Drug therapies have also been investigated as potential alternatives to surgical treatment, as listed in Table 4. These include subcutaneous calcitonin injection, α-interferon, intralesional corticosteroid injection, and ethanolamine oleate sclerotherapy.^3^ However, these methods have notable drawbacks, including prolonged treatment duration, potential need for additional surgery if ineffective, and side effects.^94,137^ Denosumab, while not yet well-described for treating PGCG, may become a treatment option for some patients.^111^

## RESULTS

### Comprehensive Management Approaches for Various Epulis Subtypes

#### Treatment modalities

As shown in Table 5, treatment approaches for the various epulis subtypes share some common principles but also require consideration of subtype-specific factors that influence treatment selection and outcomes. Table 5 provides a comprehensive comparison of treatment strategies across all epulis subtypes, highlighting both the similarities and important differences in management approaches. As demonstrated in the table, while surgical excision remains the cornerstone of treatment for most epulis variants, the specific techniques, adjunctive treatments, and management of recurrence vary significantly between subtypes. For example, giant cell epulis typically requires a more aggressive surgical approach with curettage or peripheral ostectomy to prevent recurrence, while pregnancy epulis may be managed conservatively until postpartum.

**Table 5:** Comprehensive comparison of treatment strategies for different epulis subtypes. This table provides a consolidated overview of first-line treatments, surgical considerations, alternative therapeutic approaches, and special management considerations for fibrous epulis, vascular epulis, pregnancy epulis, and giant cell epulis. Each column highlights both common approaches and subtype-specific treatment modalities that are essential for optimal clinical management.

Treatment approach	Fibrous epulis	Vascular epulis	Pregnancy epulis	Giant cell epulis
**First-line treatment**	Surgical excision with 2 mm margins	Surgical excision with 2 mm margins	Conservative management until postpartum if possible	Surgical excision with curettage or peripheral ostectomy
**Surgical considerations**	Standard excision including periosteum	Removal of underlying causes; control bleeding	Defer surgery until 2nd trimester if needed; possible postpartum resolution	Deep excision including periodontal ligament
**Laser therapy Options**	Diode, CO_2_, Er:YAG, Nd:YAG lasers	Diode, CO_2_, Er:YAG, Nd:YAG, pulsed dye lasers	Nd:YAG laser preferred during pregnancy	Diode laser, high-level laser therapy
**Minimally invasive alternatives**	May regress with removal of irritants in early lesions	Scaling and root planing for small lesions	Basic periodontal treatment; observation	Ethanolamine oleate sclerotherapy
**Sclerotherapy**	Not typically used	Pingyangmycin, ethanol, ethanolamine oleate, sodium tetradecyl sulphate	Not recommended during pregnancy	Limited evidence for effectiveness
**Adjunctive treatments**	Corticosteroid injection for recurrent cases	Corticosteroid injection for recurrent cases	Not recommended during pregnancy	Calcitonin, α-interferon in select cases
**Management of defects**	CAF, CRF, SCTG, LPF, or PRF for large defects	CAF, SCTG, LPF for aesthetic areas	Defer reconstruction until postpartum	CAF, SCTG for large defects
**Recurrence management**	Re-excision; elimination of irritants	Re-excision with wider margins; sclerotherapy	May recur in subsequent pregnancies	Re-excision with peripheral ostectomy
**Special considerations**	Lower recurrence rate than other types	Higher bleeding risk during excision	Balance maternal oral health with foetal safety	More aggressive behaviour; bone involvement


#### Prevention strategies

Prevention plays a crucial role in the comprehensive management of epulis, particularly given its strong association with local irritating factors and potential for recurrence. A multifaceted preventive approach begins with meticulous plaque control and oral hygiene maintenance. Patients should be educated on proper brushing techniques using a soft-bristled toothbrush and the importance of interdental cleaning with floss or interdental brushes to remove plaque from areas inaccessible to regular brushing. Professional dental cleanings scheduled at 3–6-month intervals are essential for removing calculus deposits that cannot be eliminated through home care alone and for early detection of developing lesions. Clinicians should conduct thorough assessments of potential chronic irritants in the oral cavity, including ill-fitting dental prostheses that may cause persistent trauma to gingival tissues. Any defective restorations with overhanging margins or rough surfaces should be promptly replaced or polished to eliminate potential irritation sites. For patients undergoing orthodontic treatment, additional preventive measures such as specialised cleaning techniques around brackets and frequent professional monitoring are recommended to prevent epulis formation. Pregnant women, who are at higher risk for vascular epulis due to hormonal changes, should receive specialised preventive counselling early in pregnancy, with emphasis on maintaining optimal oral hygiene and seeking immediate evaluation of any gingival changes. Individuals taking medications associated with gingival overgrowth, such as calcium channel blockers, cyclosporine, or phenytoin, require more frequent periodontal monitoring and may benefit from alternative medication regimens when medically feasible. Patient education about avoiding traumatic oral habits such as cheek biting or aggressive toothbrushing is an important component of prevention counselling. For patients with a history of epulis, especially those with recurrent lesions, implementing a personalised prevention programme with shorter recall intervals and targeted interventions addressing specific risk factors has shown considerable efficacy in reducing recurrence rates. The integration of these preventive strategies into routine dental practice represents a proactive approach that may significantly reduce the incidence and recurrence of epulis, ultimately improving patient outcomes and reducing the need for surgical intervention.

### Differential Diagnosis of Epulis

The diagnosis of epulis primarily relies on clinical presentation and histopathology. Epulis requires differential diagnosis from other benign and malignant gingival diseases. In addition to distinguishing between the three types of epulis mentioned above, the differential diagnosis of epulis also includes conditions such as gingival cyst, gingival hyperplasia/hyperplastic gingival inflammation, POF, haemangioma, bacillary angiomatosis, conventional granulation tissue, metastatic cancer, non-Hodgkin’s lymphoma, angiosarcoma, and Kaposi’s sarcoma.^33,73,89,112^ Table 6 provides an overview to help dentists quickly identify and differentiate between different oral lesions. Please note that the actual diagnosis also needs to be combined with a detailed history, clinical examination, and auxiliary tests as necessary.

**Table 6:** Summary of clinical presentation, histological features, common locations, radiographic features, and key differential points of different oral lesions

Lesion type	Clinical presentation	Histological features	Common location	Radiographic features	Key differential points
Fibrous epulis	Pink, smooth, sessile or pedunculated mass	Dense collagen fibre bundles	Interdental papilla	Usually no significant features	Firm texture, slow growth
Vascular epulis (PG)	Red or purple-red, easily bleeding	Capillary proliferation, inflammatory cell infiltration	Gingiva, often in anterior region	Usually no bone changes	Easy bleeding, rapid growth
Giant cell epulis	Red-purple or blue-brown, may be ulcerated	Multinucleated giant cells and mononuclear cells	Mandibular anterior region common	May show superficial bone resorption	May cause bone resorption
Gingival cyst	Blue or blue-grey translucent swelling	Cyst cavity lined with squamous epithelium	Free gingiva	No bone changes	Cystic characteristics, fluctuant on palpation
Gingival hyperplasia/Inflammatory hyperplasia	Diffuse enlargement of gingiva	Hyperplastic epithelium, increased connective tissue	Generalised or localised gingiva	No bone changes	Often drug-induced or associated with systemic conditions
Peripheral odontogenic fibroma	Firm, smooth-surfaced pink mass	Odontogenic epithelium in fibrous stroma	Attached gingiva	May show superficial bone erosion	Contains odontogenic epithelial rests
Peripheral ossifying fibroma	Pink, may be ulcerated	Fibrous tissue with osteoid or calcified material	Anterior and premolar gingiva	May show calcifications	Often has calcified or osseous tissue
Haemangioma	Purple-red, blanches on pressure	Endothelial cell proliferation, vascular space formation	Any oral site	Usually no bone changes	Blanches on pressure, deep-seated lesion
Bacillary angiomatosis	Red papules or nodules	Lobular proliferation of blood vessels, bacilli present	Any oral site	No specific features	Associated with immunosuppression, especially HIV
Conventional granulation tissue	Red, soft, granular surface	Newly formed capillaries, fibroblasts, inflammatory cells	Any site of wound healing	No specific features	Associated with healing process, resolves with time
Metastatic cancer	Irregular mass, may be ulcerated	Cancer cells similar to primary tumour	Any oral site	May show bone destruction	Rapid growth, history of primary tumour
Non-Hodgkin’s lymphoma	Firm, painless swelling	Monomorphic lymphoid cell infiltrate	Gingiva, palate common	May show bone destruction	Often part of systemic disease
Angiosarcoma	Poorly defined, purplish swelling	Malignant endothelial cell proliferation	Any oral site, palate common	May show bone destruction	Aggressive growth, poor prognosis
Kaposi’s sarcoma	Purple-red or brown patches or nodules	Spindle cells and atypical vessels	Hard palate common	May show bone erosion	Often in HIV-positive patients


When differentiating epulis from potentially malignant conditions, clinicians should be vigilant for several critical warning signs. Malignant lesions such as OSCC typically demonstrate irregular borders, indurated margins on palpation, and a non-healing ulcerated surface – features rarely seen in epulis cases. Additionally, OSCC often presents with localised paraesthesia or numbness, persistent pain, and regional lymphadenopathy, which are not characteristic of epulis. Radiographic examination is essential for thorough evaluation, as malignancies frequently show irregular, moth-eaten bone destruction with ill-defined margins, while epulis typically demonstrates either no radiographic changes or superficial, well-defined ‘cup-shaped’ resorption. Vascular malignancies like angiosarcoma can be distinguished from vascular epulis by their more aggressive growth pattern, deeper tissue invasion, and lack of response to local irritant removal. When clinical presentation raises suspicion, prompt biopsy with adequate depth and representative sampling is mandatory, as superficial biopsies may miss underlying malignant changes. For cases with atypical features or those unresponsive to conventional treatment, referral to an oral pathologist or oral oncologist should be considered without delay. To aid in clinical decision-making, we propose a concise checklist of red flags that should raise immediate suspicion of malignancy rather than epulis: 1) rapid growth over weeks rather than months; 2) persistent ulceration that fails to heal despite removal of local irritants; 3) spontaneous, recurrent bleeding not associated with trauma; 4) unexplained paraesthesia or numbness in the affected area; and 5) induration or hardness on palpation extending beyond the visible lesion. The presence of two or more of these warning signs warrants urgent biopsy and potential referral to an oral and maxillofacial surgeon or oral oncologist for comprehensive evaluation.

**Fig 3:**
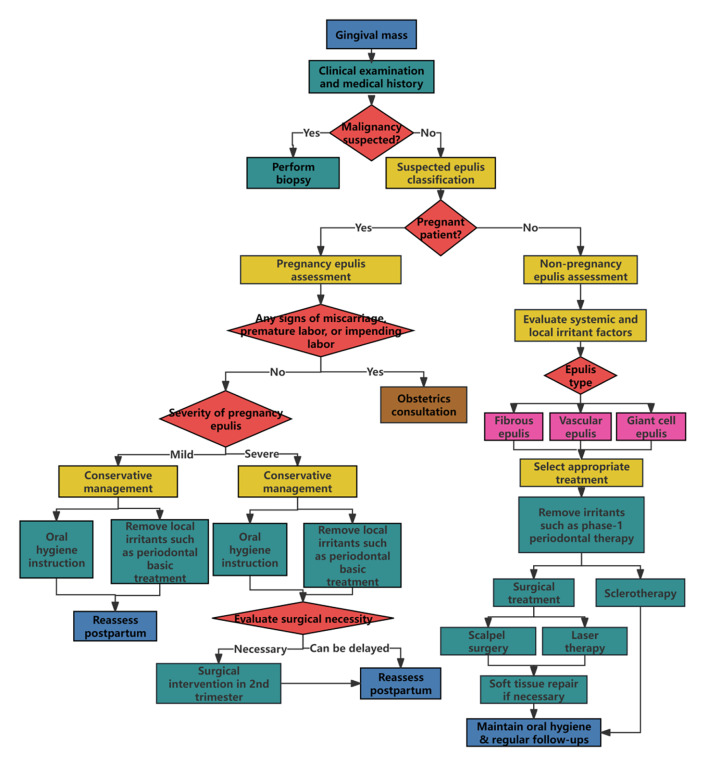
Proposal of a flowchart for the diagnosis and treatment of epulis.

The clinical presentations of various gingival lesions can be very similar, often presenting diagnostic challenges. Clinically, epulis needs to be differentiated from other benign and malignant gingival lesions. There is a high consistency between clinical diagnosis and histopathological diagnosis of epulis (overall 82.5%), especially for IFH (96%). OPG (68%), POF (57%), and PGCL (47%) show moderate correlation, while GCF has an extremely low correlation (7%).61 Therefore, understanding the clinical characteristics of epulis aids in preliminary diagnosis. For suspicious lesions, radiological examinations, blood screening, and biopsy should be combined, relying on histopathology for definitive diagnosis. For oral clinicians, it is crucial to be able to recognise their clinical characteristics, differentiate between benign and malignant diseases, and identify when a biopsy is necessary. This aids in assessing the patient’s condition, early diagnosis, appropriate management, and treatment planning. As shown in Figure 3, a flowchart summarising the diagnosis and treatment of epulis has been compiled, aiming to provide a reference for clinical practice.

### Genetic and Molecular Mechanisms of Epulis

The pathogenesis of epulis involves complex interactions between genetic factors, molecular signalling pathways, and environmental stimuli that collectively contribute to the development and progression of these lesions. One significant molecular mechanism implicated in fibrous epulis is the upregulation of the aryl hydrocarbon receptor (AhR), which has been found to be significantly overexpressed in fibrous epulis at both mRNA and protein levels. AhR functions as a regulatory factor in inflammation and tissue homeostasis, playing a crucial role in the development of epulis by promoting inflammation and inhibiting apoptosis. Studies have demonstrated that suppression of AhR using siRNA in human periodontal ligament cells and gingival fibroblasts leads to decreased expression of proinflammatory cytokines and apoptosis-related genes, which suggests that AhR activation contributes to persistent inflammation and cellular survival in epulis tissues.^181^ These molecular findings have significant clinical implications, as they help explain why some epulis lesions persist despite removal of local irritants and may contribute to the varying recurrence rates observed clinically among different epulis subtypes. Understanding the role of AhR could potentially lead to novel therapeutic approaches targeting this pathway, particularly for recurrent cases where traditional surgical excision has proven ineffective.

Beyond genetic factors, several intracellular signalling pathways play crucial roles in the pathogenesis of epulis, particularly in fibrous variants. The RAS-PI3K-AKT-NF-κB pathway has emerged as a central regulator in fibrous epulis development, where it transcriptionally controls the expression of anti-apoptotic genes from the *BCL2* and *IAP* families. RNA sequencing and qRT-PCR analyses have revealed significant upregulation of key components of this pathway, including *SOS1*, *HRAS*, *PIK3CA*, *AKT3*, and various NF-κB pathway elements.^92^ This pathway activation results in hyperproliferation of gingival cells and inhibition of apoptosis, directly contributing to the persistent growth characteristics observed clinically in fibrous epulis lesions.^91^ Interestingly, while the PI3K/AKT pathway typically promotes autophagy in many cellular contexts, studies indicate that autophagy is not significantly involved in fibrous epulis pathogenesis, as evidenced by the lack of LC3 conversion, suggesting a selective activation of proliferative and anti-apoptotic mechanisms rather than cellular recycling processes.^91^ Additionally, the Eph/ephrin signalling pathway, though more extensively studied in cancer and inflammatory conditions, likely contributes to epulis development through its effects on cell adhesion, migration, and angiogenesis.^51^ Specifically, EphA2 receptor signalling influences both angiogenic processes and inflammatory responses that could be relevant to the vascular and inflammatory characteristics observed in various epulis subtypes.^101,179^ The identification of these specific signalling pathways has significant therapeutic implications, as they represent potential targets for molecular-based interventions, particularly for recurrent or treatment-resistant cases. For instance, small molecule inhibitors targeting the RAS-PI3K-AKT-NF-κB pathway or AhR antagonists could potentially serve as adjunctive treatments to conventional surgical management, offering more precise approaches to disrupting the molecular mechanisms driving epulis formation and persistence.

Beyond genetic factors and signalling pathways, growth factors and extracellular matrix proteins also contribute significantly to epulis development. Basic fibroblast growth factor (bFGF) has been identified as an important mediator in the pathogenesis of epulis, particularly in vascular subtypes. In the granulation tissue of epulis, bFGF is primarily produced and released by macrophages and mast cells into the extracellular matrix, where it participates in angiogenesis and tissue remodelling processes.^125^ Additionally, tenascin-C, a functional protein associated with connective tissue organisation and cell migration, shows increased expression in epulis, especially around blood vessels with plump endothelial cells and in areas of keratinocyte migration.^120^ The interaction between these growth factors and the extracellular matrix creates a microenvironment conducive to the persistent growth characteristics observed clinically in epulis lesions. These insights have direct clinical relevance, as they help explain the vascular nature of certain epulis subtypes and suggest potential therapeutic targets. For instance, anti-angiogenic agents that inhibit bFGF signalling could be explored as adjunctive treatments for vascular epulis, which currently shows high recurrence rates. Additionally, the connective tissue growth factor (CTGF/CCN2) has been found to be highly expressed in both epithelial and connective tissues in gingival fibromatosis lesions, suggesting that epithelial-mesenchymal interactions may promote gingival fibrosis,^97^ which could serve as another target for molecular-based therapies.

The host-microbial interaction represents another dimension of epulis pathogenesis with significant clinical implications. Although epulis is primarily considered a reactive proliferation, bacterial colonisation plays a role in its development. Gingival epithelial cells possess sophisticated defence mechanisms against bacterial invasion, including danger molecule signalling pathways and antimicrobial effector molecules. However, certain adaptive bacteria, such as *Porphyromonas gingivalis*, can target these defence pathways, leading to persistent bacterial presence and chronic inflammation.^109^ The immunohistochemical profile of congenital granular cell epulis, showing immunoreactivity for neuron-specific enolase (NSE) and vimentin but lacking reactivity for S-100 protein, laminin, and chromogranin, provides additional insights into the cellular origins and molecular characteristics of these lesions.^50,167^ From a clinical perspective, these findings support the current practice of combining surgical excision with thorough periodontal therapy and suggest potential for personalised medicine approaches based on genetic and epigenetic profiles of individual patients. Future molecular-targeted approaches might include antimicrobial peptides specifically designed to disrupt bacterial colonisation processes or immunomodulatory agents that enhance epithelial defence mechanisms without promoting hyperproliferative responses, thereby translating our molecular understanding into improved diagnostic tools and treatment outcomes for patients with epulis.

The recurrence patterns of epulis vary significantly across subtypes, with reported rates differing substantially depending on the histological variant. Multiple factors contribute to recurrence risk, extending beyond incomplete surgical excision. Molecular and genetic factors play a crucial role, as demonstrated by studies showing that AhR overexpression and RAS-PI3K-AKT-NF-κB pathway activation promote persistent inflammation and inhibit apoptosis in epulis tissues. Hormonal influences represent another significant risk factor, particularly evident in the recurrence of pregnancy epulis during subsequent pregnancies due to fluctuating oestrogen and progesterone levels. Surgical technique significantly impacts recurrence rates, with evidence suggesting that excision including the periosteum and periodontal ligament reduces recurrence, as does peripheral ostectomy in giant cell epulis cases. Patient-specific factors including age, immunological status, and systemic conditions may also modify recurrence risk, though these relationships require further investigation through prospective studies with standardised surgical protocols and long-term follow-up.

## CONCLUSION

### Future Outlook

The field of epulis research is poised for significant advancements in the coming years. One of the most promising areas is the application of molecular biology and genetic techniques to elucidate the pathogenesis of different types of epulis. Advanced genomic and proteomic analyses may reveal specific genetic markers or molecular pathways associated with epulis development and progression. This knowledge could lead to the development of targeted therapies and personalised treatment approaches. Moreover, the identification of genetic predispositions to epulis could enable early intervention strategies for high-risk individuals.

Another exciting avenue for future research is the exploration of novel treatment modalities. While current treatments primarily focus on surgical excision and laser therapy, emerging technologies such as photodynamic therapy, nanotechnology-based drug delivery systems, and immunomodulatory approaches hold promise for more effective and less invasive management of epulis. Additionally, there is a growing interest in the potential use of stem cell therapy and tissue engineering techniques for the regeneration of gingival tissues affected by epulis. Large-scale, multicentre clinical trials are needed to evaluate the long-term efficacy and safety of these innovative approaches compared to conventional treatments.

Furthermore, the role of systemic factors in epulis development warrants deeper investigation. Future studies should focus on elucidating the complex interplay between local irritants, hormonal influences, and systemic conditions in the aetiology of epulis. This could lead to more comprehensive prevention strategies and improved management of epulis in patients with underlying systemic disorders. Lastly, the development of standardised, evidence-based guidelines for epulis management, particularly for special patient populations such as pregnant women or individuals with systemic diseases, represents a critical goal for future research efforts. These guidelines would greatly enhance clinical decision-making and improve patient outcomes across diverse healthcare settings.

Epulis remains a significant challenge in oral health, requiring a comprehensive clinical approach based on precise diagnosis and subtype identification. While surgical excision remains the primary treatment modality, clinicians should tailor their approach to specific epulis subtypes: incorporating sclerotherapy for recurrent vascular lesions, applying peripheral ostectomy for giant cell variants, and considering laser therapy for aesthetically sensitive areas. The management strategy must extend beyond initial intervention to include thorough excision of the periodontal ligament, elimination of local irritants, implementation of rigorous oral hygiene protocols, and scheduled follow-ups at 3–6-month intervals for high-risk patients. Prevention through patient education and professional periodontal maintenance represents a critical component of successful management, particularly for pregnancy-associated cases and patients with recurrent lesions. The strong link between periodontal health and epulis recurrence underscores the importance of integrating epulis management into comprehensive oral healthcare protocols. Future research addressing identified knowledge gaps is essential for advancing epulis management. Particularly needed are studies exploring molecular pathogenesis to develop targeted therapies, large-scale epidemiological investigations across diverse populations, controlled trials comparing treatment modalities with standardised protocols, and investigations into the relationship between systemic conditions and epulis development. As this research progresses, it will enable the development of evidence-based standardised guidelines for various epulis subtypes, ultimately improving long-term outcomes for affected patients.
